# Two Uncommon Sites of Metastasis: Breast and Hypophysis Metastases of Head and Neck Adenoid Cystic Carcinoma Detected by FDG PET/CT

**DOI:** 10.4274/mirt.06025

**Published:** 2017-10-02

**Authors:** Evrim Sürer Budak, Şenay Yıldırım, Sevim Yıldız, Ali Ozan Öner, Şeyda Gündüz

**Affiliations:** 1 Antalya Training and Research Hospital, Clinic of Nuclear Medicine, Antalya, Turkey; 2 Antalya Training and Research Hospital, Clinic of Pathology, Antalya, Turkey; 3 Antalya Training and Research Hospital, Clinic of Radiology, Antalya, Turkey; 4 Afyon Kocatepe University Faculty of Medicine, Clinic of Nuclear Medicine, Afyon, Turkey; 5 Antalya Training and Research Hospital, Clinic of Medical Oncology, Antalya, Turkey

**Keywords:** PET/CT, adenoid cystic carcinoma, Breast, hypophysis, metastasis

## Abstract

Adenoid cystic carcinoma (ACC) is a rare epithelial malignancy arising from secretory glands, particularly the salivary glands. It tends to invade nerves and has a high potential for distant hematogenous metastasis, especially to the lungs, bone, liver and brain. The breast and hypophysis are not common sites of ACC metastatic disease. Herein, we report a case of ACC of the head and neck region with two unusual sites of metastases, the hypophysis and breast.

## Abstract (tr)

Adenoid kistik karsinom (AKK), sekretuar glandlardan özellikle de tükrük bezlerinden köken alan nadir bir epitelyal tümördür. Sinir invazyonu sıktır ve özellikle akciğer, kemik, karaciğer ve beyin gibi organlara uzak hematojen metastaz yapma potansiyeli yüksektir. Ancak, meme ve hipofiz AKK için tipik metastaz alanları değildir. Bu çalışmada, hipofiz ve meme gibi iki olağan dışı alana metastazı olan baş-boyun AKK olgusunu sunmaktayız.

## Figures and Tables

**Figure 1:**
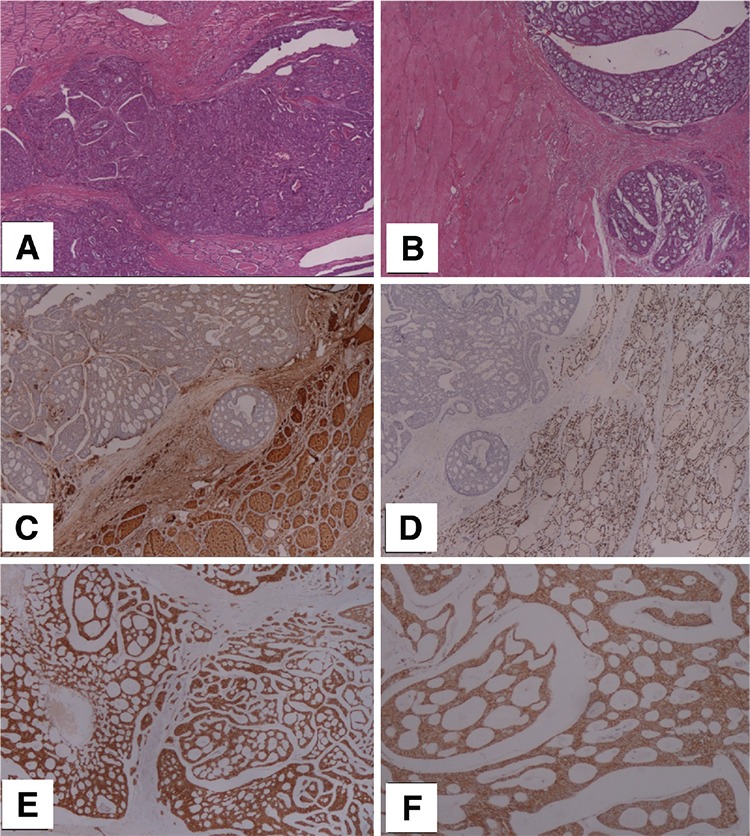
A 49-year-old woman presented with a left cervical mass. The ultrasound examination revealed a gross mass adjacent to the thyroid gland and she underwent total excision of the mass along with total thyroidectomy. Histopathologic examination of the 9.5x10 cm mass revealed adenoid cystic carcinoma (ACC) infiltrating both the thyroid and adjacent smooth muscle tissue. (A, B): Hematoxylin and eosin (H&E) staining of the primary tumor: Tumor cells were arranged in cord-like or acinar-like by atypical hyperplastic epithelial cells forming a cribriform and tubular pattern with a mucoid luminal material (x40). (C, D): Tumor cells were negative for thyroglobulin (C) and TTF-1 (D) excluding thyroid origin (x40). Tumor cells were immune-positive for CD-117 (E) indicating ductal origin and E-cadherin (F) (x100). She was accepted as ACC of the head and neck region. The patient was referred to FDG PET/CT scanning for initial staging. PET/CT images revealed multiple hypermetabolic lung metastasis and a mild FDG uptake in the operation region secondary to surgery. She received radiotherapy and chemotherapy, and was stable for about 20 months follow-up

**Figure 2:**
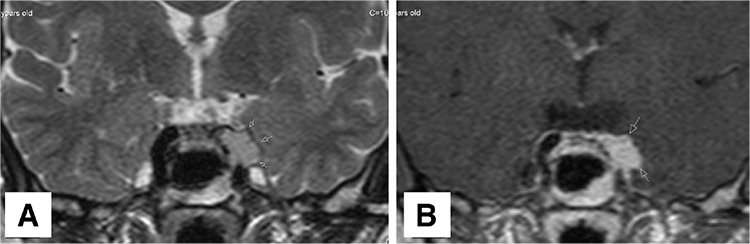
(A, B) Twenty months after the initial diagnosis, the patient developed sudden vision loss. The cranial magnetic resonance imaging T2 and post-contrast T1 weighted coronal images revealed a mildly T2 hyper-intense and homogenously enhancing left parasellar mass compatible with metastasis. The patient received radiotherapy. Pituitary metastasis is very rare, accounting for only about 1% of pituitary surgeries (1). In the literature, although extraordinary metastasis sites of ACC were reported including the kidney (2) and vertebrae (3), there are only two cases reporting hypophysis metastasis (4,5)

**Figure 3:**
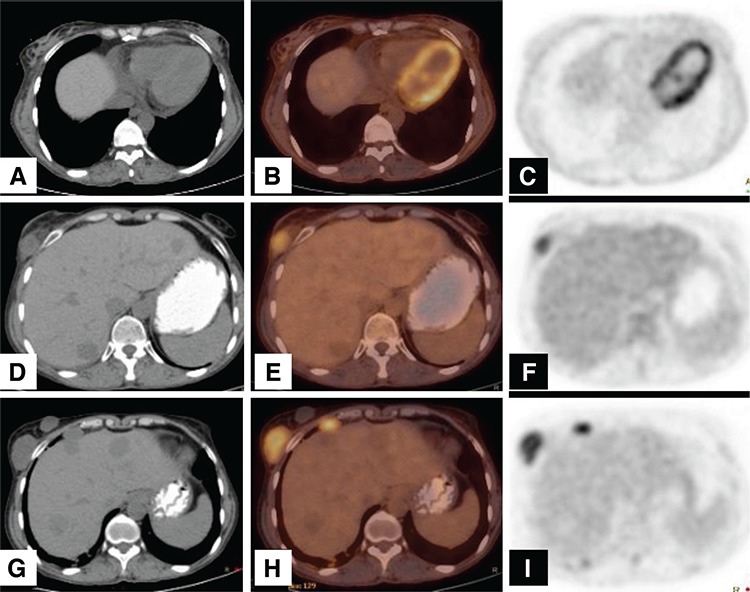
The PET/CT images performed for response evaluation at the 24th month after initial diagnosis showed two new lesions in the right breast. The interval between the last two PET/CT imaging was about one year, and these breast lesions were new findings. (A, B, C): The initial FDG PET/CT images with no lesion both anatomically and metabolically in the breast tissue. (D, E, F): The second year FDG PET/CT examination revealed an unexpected, lobulated hypermetabolic solid mass measuring 19x30 mm with a SUVmax of 5.2 in the lower outer quadrant of the right breast. There was also a smaller non-metabolic lesion next to this lesion. The patient underwent incisional biopsy, the histopathologic examination revealed ACC in the lateral lesion while the other one was composed of necrotic material. The histology and immunophenotyping of the breast ACC is similar to that of the salivary gland. Therefore, it is difficult to differentiate metastatic disease from a primary breast ACC by pathologic examination. The diagnosis is usually based on clinical behavior of the tumor. In our case, based on the clinical findings (sudden onset in one year), it was diagnosed as metastasis from the primary tumor. Hypermetabolic right parasternal, anterior diaphragmatic/paracardiac lymph nodes were also detected in the same examination. (G, H, I): In the following FDG PET/CT, her metastatic breast lesion progressed anatomically (24x35 mm) while remaining metabolically stable (SUVmax: 5.6). Anterior mediastinal, paracardiac, parasternal and right anterior mediastinal lymph nodes were also detected as new findings while the existing lung metastasis were progressing. (D, G): The patient also had multiple hypodense stable lesions in the liver that did not show any FDG uptake, compatible with hemangiomas. The patient’s chemoradiotherapy is still on-going in the third year of diagnosis. In the consecutive follow-up PET/CT images performed at 4 month intervals, her metastatic lung lesions, lymph nodes and breast lesion all have been progressing both in dimension and metabolically. Breast metastasis from extra-mammary neoplasms is very rare, with a reported incidence of 0.5-3% (6). Also, the incidence of unexpected breast focal uptake in 18F-DG-PET/CT is reported as 0.36-1.12% (7,8,9). The rate of malignancy in incidental FDG-avid breast foci was reported in a range of 37.5-83% (10,11,12). Under these circumstances, it can be concluded that focal FDG-avid breast lesions need further evaluation, especially in cases with known malignancies

**Figure 4:**
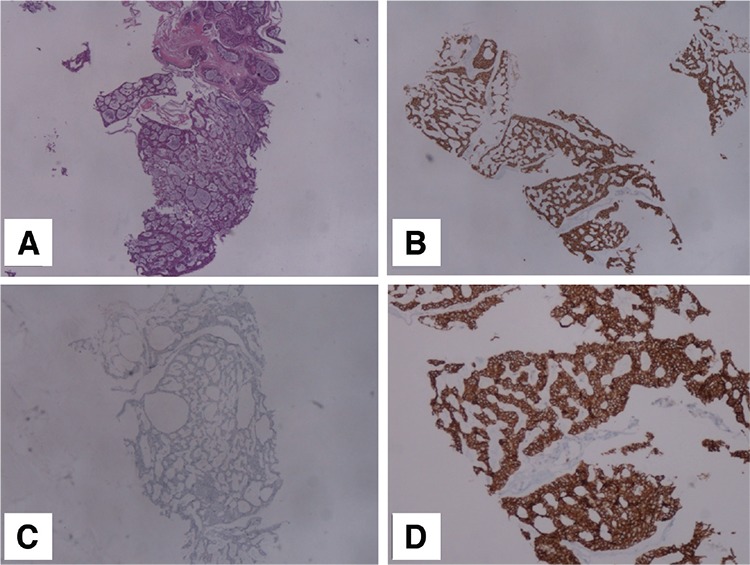
Histopathologic examination of the breast lesion revealed ACC. (A): Invasive tumor cells forming cylindromatous nodules and glandular spaces with basophilic secretion (x40) (B): CD-117 positivity in tumor cells (x40) (C): Tumor cells were negative for Estrogen receptor (x40) (D): Tumor cells were immune-positive for E-cadherin (x100)
